# Auditory Rhythm Encoding during the Last Trimester of Human Gestation: From Tracking the Basic Beat to Tracking Hierarchical Nested Temporal Structures

**DOI:** 10.1523/JNEUROSCI.0398-24.2024

**Published:** 2024-12-23

**Authors:** Bahar Saadatmehr, Mohammadreza Edalati, Fabrice Wallois, Ghida Ghostine, Guy Kongolo, Erica Flaten, Barbara Tillmann, Laurel Trainor, Sahar Moghimi

**Affiliations:** ^1^Inserm UMR1105, Groupe de Recherches sur l’Analyse Multimodale de la Fonction Cérébrale, CURS, Amiens Cedex 80036, France; ^2^Inserm UMR1105, EFSN Pédiatriques, CHU Amiens sud, Amiens Cedex 80054, France; ^3^Department of Psychology, Neuroscience and Behaviour, McMaster University, Hamilton, Ontario L8S 3L8, Canada; ^4^Laboratory for Research on Learning and Development, LEAD – CNRS UMR5022, Université de Bourgogne, Bourgogne-Franche Comté, Dijon 21000, France; ^5^McMaster Institute for Music and the Mind, McMaster University, Hamilton, Ontario L8S 3L8, Canada; ^6^Rotman Research Institute, Baycrest Hospital, Toronto, Ontario M6A 2E1, Canada

**Keywords:** electroencephalography, music, neural synchronization, premature human brain

## Abstract

Rhythm perception and synchronization to periodicity hold fundamental neurodevelopmental importance for language acquisition, musical behavior, and social communication. Rhythm is omnipresent in the fetal auditory world and newborns demonstrate sensitivity to auditory rhythmic cues. During the last trimester of gestation, the brain begins to respond to auditory stimulation and to code the auditory environment. When and how during this period do the neural capacities for rhythm processing develop? We conducted a cross-sectional study in 46 neonates (24 male) born between 27 and 35 weeks gestational age (wGA), measuring their neural responses to auditory rhythms with high-density electroencephalography during sleep. We developed measures to evaluate neural synchronization to nested rhythmic periodicities, including the fast isochronous beat and slower metrical (beat grouping) structures. We show that neural synchronization to beat and meter becomes stronger with increasing GA, converging on small phase differences between stimulus and neural responses near term, similar to those observed in adults. Dividing the cohort into subpopulations born before and after 33 wGA revealed that both younger and older groups showed neural synchronization to the fast periodicity related to the isochronous beat, whereas only the older group showed significant neural synchronization to the slower meter frequencies related to beat groupings, suggesting that encoding of nested periodicities arrives during late gestation. Together, our results shed light on the rapid evolution of neural coding of external hierarchical auditory rhythms during the third trimester of gestation, starting from the age when the thalamocortical axons establish the first synapses with the cortical plate.

## Significance Statement

The ability to process auditory rhythms is of great neurodevelopmental importance as it underlies the development of language and music processing. In a cross-sectional electroencephalography experiment, we found that the premature brain begins to code the isochronous beat at the beginning of the third trimester of gestation. Neural synchronization to rhythmic periodicities improves with increasing gestational age, and before term, neural oscillations entrain to multiple periodicities in auditory rhythms, similarly to human adults. This provides the first evidence for neural coding of rhythm during the very early stages of auditory cortical neurodevelopment, when the neural response can first be recorded in humans from the time when the first thalamic afferents enter the cortical plate, approximately 28 weeks gestational age.

## Introduction

Musical rhythms (and to a lesser extent speech) can be perceived to have an underlying regular periodic structure, including a basic isochronous beat level, to which people can entrain their movements and predict the timing of the next beat. While the perceived beat (i.e., sequence of regularly felt pulses) is derived from regularities in the rhythm, it is not completely given by the rhythm, as rhythms usually contain event onset-to-onset durations of different lengths (often in ratios of 1:2, 1:3, or 2:3) as well as “rests” or silences on which people still feel a beat. Furthermore, in addition to the isochronous derived beat level, listeners typically perceive a metrical hierarchy of beat tempos (levels), typically consisting of groupings of two (as in a march) or three (as in a waltz) beats of the level below. Even in the case of relatively complex rhythms, adults can extract rhythmic periodicities, perceive a metrical hierarchy, and synchronize their behavior to it (Nozaradan et al., 2012; Lenc et al., 2020; Stupacher et al., 2022a,b). Furthermore, a particular rhythm can be metrically ambiguous, that is, it can be perceived as having different meters; for example, a six-beat repeating rhythm can be perceived as two groups of three beats or as three groups of two beats (Cirelli et al., 2016; Flaten et al., 2022).

Deriving and perceiving real-time temporal organization in rhythmic patterns is crucial for both music and language perception as the sound events in these communication channels occur rapidly and, once sounded, cannot be resampled. Rhythm perception and synchronization to periodicity at different tempos or timescales are also of high importance from the developmental point of view (Lense et al., 2021; Snyder et al., 2024) for language development (Goswami, 2022), music behaviors (Trainor and Hannon, 2013), bonding, and social interaction early in development (Cirelli et al., 2018; Savage et al., 2021; Nguyen et al., 2023). Neural tracking of beat hierarchies has been shown in infants between 5 and 7 months after birth (Cirelli, et al., 2016; Lenc et al., 2023). However, it is yet not known when the neural capacities for coding these temporal hierarchies first appear and how they develop. Near term or at term birth, infants possess a wide range of auditory processing abilities. Some of these capacities are universal and relate to the neural coding of the acoustic characteristics of sound (Nazzi et al., 1998; Ramus et al., 2000; Dehaene-Lambertz and Pena, 2001; Mahmoudzadeh et al., 2013), while others reflect effects of specific prenatal in utero auditory experience (Granier-Deferre et al., 2011; Moon, 2017; Mariani et al., 2023).

Processing the information arriving from the auditory environment requires the development of primary auditory and associative cortical networks subsequent to the peripheral sensory organs and subcortical pathways. Auditory perception develops progressively during the last trimester of pregnancy and continues to refine for many years after birth. The cochlea becomes functional at approximately 24–26 weeks gestational age (wGA) in humans (Pujol et al., 1998), and the transmission of sound signals through the subcortical nuclei of the auditory pathways follows, as suggested in animal models (Geal-Dor et al., 1993; Babola et al., 2018; Makarov et al., 2021). Between 26 and 28 wGA in humans, the thalamocortical afferents invade the cortical plate of their target areas, where the first synapses appear. Between 28 and 30 wGA, the thalamocortical axons establish synapses with cortical plate layer IV neurons and become (at least partly) functionally sensory-driven (Kostović and Judaš, 2010; Molnár et al., 2019). In agreement with the immature state of structural neurodevelopment, the latencies of electrophysiological responses to auditory stimulation are very long, and their amplitudes are very low at approximately 28–29 wGA (Rotteveel et al., 1987; Daneshvarfard et al., 2020). By 32–33 wGA, the fetal auditory system has become more mature, and the overall morphology and latency of cortical evoked potentials approach those of a full-term newborn.

From a functional perspective, it is not known yet how and when the neural coding of basic acoustic features gradually evolves into the elaborated networks supporting the auditory experiences observed in adults (Lenc et al., 2020; Zalta et al., 2023). While rhythmic coding is likely fundamental in this development, it is yet to be investigated whether the developing brain extracts the underlying beat and hierarchical metrical structure of rhythms as soon as the auditory cortex is connected to the outside world through thalamic afferents, which occurs near the beginning of the last trimester of pregnancy or whether this neural capacity arrives later in the third trimester of gestation.

**Figure 1.**
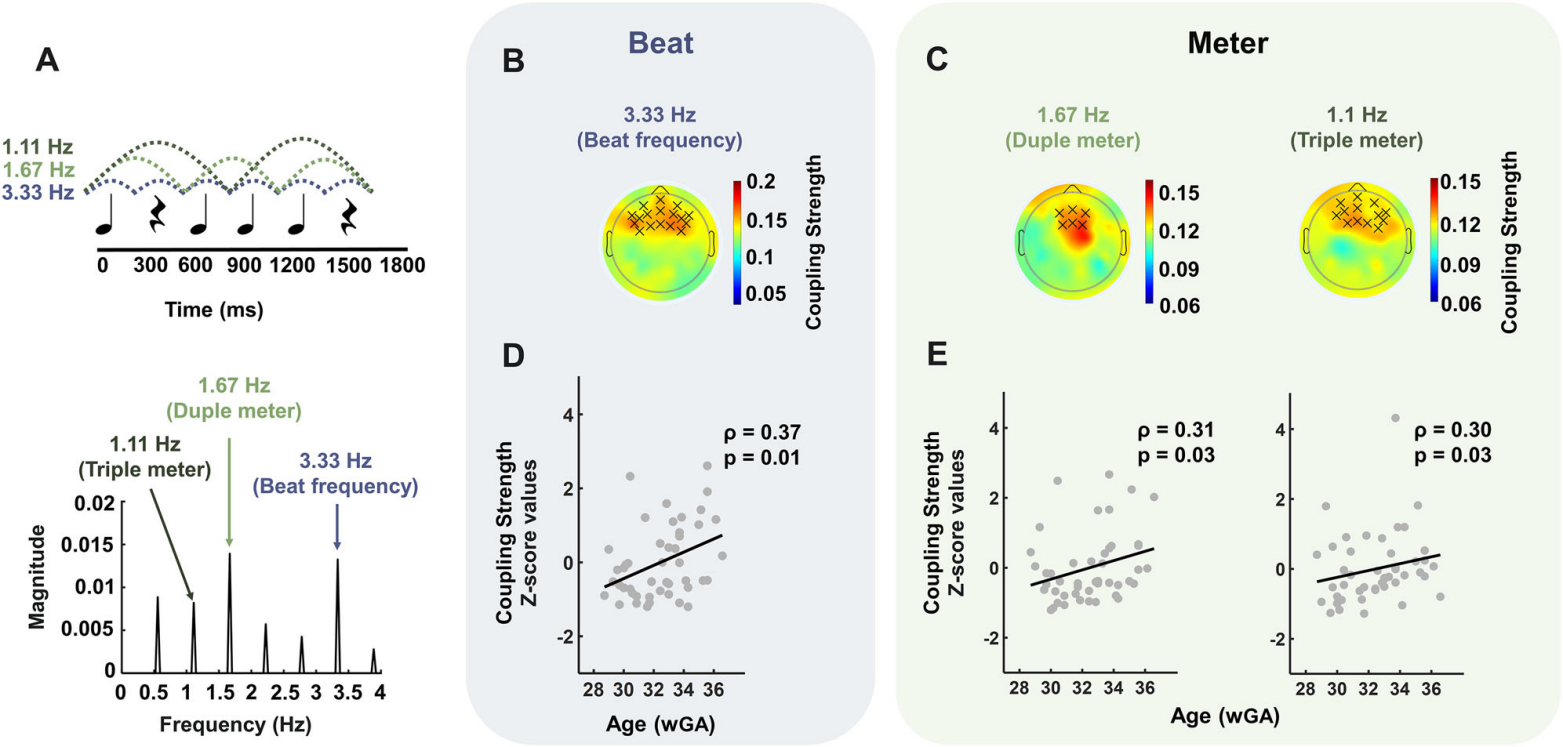
Development of neural synchronization to the rhythmic hierarchy. ***A***, The auditory sequence. The rhythmic pattern is composed of 300 ms duration tones and rests (top). The dashed lines superimposed on the figure show the beat and metrical levels. The frequency spectra of the stimulus sound envelope are shown below the rhythmic pattern. The topographical scalp distributions of the grand average SI absolute values for both beat- (***B***) and meter-related (***C***) frequencies. The black crosses overlaid on the topographical distributions represent clusters where the coupling strength was significant: a frontocentral cluster for the beat frequency (***B***, *p* = 0.0014), a frontocentral cluster for the duple meter frequency (***C***, left *p* = 0.032) with a relatively more focalized topographical distribution, and a frontocentral cluster for the triple meter frequency (***C***, right, *p* = 0.036). ***D***, ***E***, Relation between gestational age and neural response to the rhythmic hierarchy. Scatter plots demonstrating significant positive correlation in the full cohort average *z*-scored SI values with gestational age for the beat (***D***, Spearman correlation, *ρ* = 0.37, *p* = 0.01), duple meter (***E***, left, Spearman correlation, *ρ* = 0.31, *p* = 0.03), and triple meter (***E***, right, Spearman correlation *ρ* = 0.30, *p* = 0.03) frequency, averaged across the detected clusters presented in (***B***, ***C***). The black solid line shows a linear fit to the data.

Using frequency tagging and brain–stimulus synchronization measures, we have recently shown that the brains of premature neonates born between 30 and 34 wGA (*N* = 19) track beat and meter frequencies in auditory rhythms (Edalati et al., 2023). However, it is not yet known how early the premature brain starts to structure its auditory environment and code hierarchical temporal regularities. This investigation requires a population that spans over a larger preterm developmental period. We here report a cross-sectional study with 46 premature neonates (considered clinically “normal” for their gestational age), born during the third trimester of gestation between 27 wGA and 35 wGA. Our aim was to investigate how the encoding of the metrical hierarchy, including the basic beat and higher grouping levels, develops across this developmental period. Approximately 1 week after birth, we recorded EEG to evaluate their neural activity during sleep in response to an auditory rhythmic sequence. We used the repeating six-beat stimulus of Flaten et al. (2022) (Fig. 1*A*). This repeating six-beat rhythm is ambiguous in containing evidence for both two-beat (three groups of two beats, hereafter referred to as duple meter) and three-beat (two groups of three beats, hereafter triple meter) groupings. In the present study, we exploited the spatial and temporal richness of premature EEG by combining a dense scalp coverage (custom design net with 64–124 electrodes usable with infants as young as 28 wGA) with stimulus–brain synchronization analysis measures. The perception of the basic beat underlying our experimental stimulus requires encoding the temporal regularity of successive events (tones and rests), whereas meter perception requires encoding the slower periodicities at groupings of two or three beats. We tested the hypothesis that neural coding of the metrical hierarchy develops with age and therefore becomes more robust over the third trimester of gestation. More precisely, we postulated that younger-born premature infants would show the encoding of the basic beat by neurally synchronizing to the faster periodicity in the stimulus, whereas the neural encoding of higher levels of the metrical hierarchy would develop and become robust later in the course of the third trimester of gestation as the auditory and associative cortical networks mature.

## Materials and Methods

### Participants

Forty-six healthy premature neonates participated in our study, including 24 males, with a mean gestational age at birth of 31.22 ± 2.01 wGA (age range at birth 27–35 wGA, mean recording age of 32.46 ± 2.13 wGA, with testing occurring 8.5 ± 2.64 d after birth for all participants). EEGs were recorded during sleep at the neonatal intensive care unit of the Amiens University Hospital in France. In brief, all neonates had appropriate birth weight, size, and head circumference for their gestational age and normal auditory and clinical neurological assessments. None were considered to be at risk of brain damage. Both parents were informed about the study and provided their written informed consent. The ethics committee (Comité de Protection des Personnes Ouest I) approved the study (ID-RCB: 2019-A01534-53). Participants were subsequently divided into two groups (younger preterm, age range of 28–32 wGA, <33 wGA, *n* = 23; older preterm, age range of 33–36 wGA, ≥33 wGA, *n* = 23) based on their age at the time of recording.

### Auditory stimuli and the experimental procedure

The auditory stimulus was a rhythmic sequence, specifically in the pattern of tone, rest (silence), tone, tone, tone, and rest, originally developed by Phillips-Silver and Trainor (2005) and used by Flaten et al. (2022). Briefly, onset-to-onset intervals between successive tones were either 300 or 600 ms, such that successive tone or silence onset-to-onsets were 300 ms, resulting in a total duration of 1,800 ms for the entire pattern. On each trial, the rhythmic pattern was repeated 20 times in a row, resulting in 36-s-long trials. Tones were synthesized with piano, vibraphone, or guitar timbres using the Apple program GarageBand (Flaten et al., 2022) to ensure the generalization of results across timbres. Vibraphone and guitar tones were synthesized with a pitch of 440 Hz (corresponding to the note A), while piano tones were synthesized at three pitches (440, 554.37, and 659.26 Hz, corresponding to the notes A, C#, and E, respectively). Pitch and timbre were consistent within a trial but varied between trials, as indicated below. The rhythmic patterns were created using the open-source software Audacity 2.2.2 and exported as WAV files. This rhythmic stimulus contained energy peaks at the beat (3.33 Hz), duple meter (2 × 300 ms, 1.67 Hz), and triple meter (3 × 300 ms, 1.11 Hz) frequencies (Fig. 1*A*).

Each experimental session started with a 20 min period of silence, during which we recorded the neonate's spontaneous neural activity for clinical evaluation and as a baseline condition for future analysis (data not shown here). Subsequently, the trials were presented to the sleeping neonates in six blocks, separated by periods of silence, randomly ranging from 2 to 3 s. Each block consisted of nine trials, presented without breaks between trials, making its duration equal to 5 min and 24 s. Within each block, either the pitch (using piano tones only) or timbre (using vibraphone, guitar, or piano timbres with a pitch of A) of the tones changed from one trial to the next in pseudorandom order, such that the same pitch or timbre was not presented on successive trials. The entire duration of the stimulus presentation was ∼32 min and 36 s. The stimuli were delivered via loudspeakers placed at the neonates' feet, with a sound level of 65 dB SPL using Psychtoolbox for MATLAB (Kleiner et al., 2007). Recordings were paused when the infants woke up, began to cry, or exhibited movement and started again once the infant became calm.

Given that we averaged the EEG data across blocks with different timbres in order to increase the signal-to-noise ratio, to characterize the stimulus, we also averaged the stimuli across the blocks. We then extracted the stimulus temporal envelope using the Hilbert transform. The obtained waveforms were then transformed into the frequency domain using a discrete Fourier transform, resulting in the frequency spectra of the acoustic energy of the stimulus. As depicted in Figure 1*A*, clear peaks were observed at the beat (3.33 Hz), duple (1.67 Hz), and triple (1.11 Hz) meter frequencies, as well as their superharmonic (2.22, 2.78 Hz) and subharmonic (0.56 Hz) frequencies.

### Data acquisition and preprocessing

EEG signals were recorded using both a 124-channel and a 64-channel HydroCel Geodesic Sensor Net (depending on the head circumference of the neonates) with an Electrical Geodesic NetAmps 200 amplifier passing a digitized signal to Electrical Geodesics Net Station software (version 5). The EEG was digitized at a 1,000 Hz sampling rate, with a Cz vertex electrode as a reference. The recorded signals were analyzed with MATLAB software (MathWorks) using FieldTrip (Oostenveld et al., 2011), EEGLAB (Delorme and Makeig, 2004), and custom MATLAB functions and codes. We applied a two-pass 0.5–15 Hz finite impulse response filter (order, three cycles of the low-frequency cutoff) from the EEGLAB toolbox to remove low- and high-frequency artifacts from the EEG signals. The data were then down-sampled to 512 Hz. Due to the different number of electrodes used for the recordings (64 or 124), 124-electrode recordings were spatially down-sampled to a 64-electrode montage for full cohort analyses. Electrodes located in the outer ring were excluded from further analyses due to poor signal-to-noise ratio. Next, the data were preprocessed using the MATLAB scripts of the APICE EEG preprocessing pipeline (Fló et al., 2022b). Finally, the data were re-referenced to the average of all electrodes.

### Stimulus–brain synchronization

To evaluate brain synchronization (coupling strength) to the rhythmic sequences at beat and meter frequencies, we computed the phase difference between narrow-band filtered neural oscillations and the beat and metrical periodic dynamics. Toward this, we approximated the beat, duple, and triple periodic dynamics with sinusoidal oscillations at 3.33, 1.67, and 1.11 Hz, respectively. Each cycle of the sinusoid represented the duration between two successive corresponding events within the rhythmic sequence (a tone or silence for beat; first, second, and fourth tones for duple meter; and first and third tones for triple meter), with the peak aligned with the event onsets. To avoid the impact of rapid fluctuations on the temporal evolution of the phase time series, we narrow-band filtered (zero-phase, FIR, bandwidth 0.2 Hz) each EEG trial around the frequencies corresponding to beat and meter. Next, we applied the Hilbert transform to both the narrow-band filtered EEG signal and the corresponding sinusoidal signal to calculate the phase time series. We eliminated 2 s from the beginning and the end of each trial. Next, we calculated the synchronization index (SI) between the two-phase time series for each time sample and then averaged over time samples and trials at each electrode location for each participant. SI is a complex value, with the absolute value indicating the strength of coupling between neural oscillations and periodicities in the stimulus, and the angle representing the “preferred phase” of coupling (Staresina et al., 2015; Gonzalez et al., 2018). The SI was calculated as

$$\mathrm{SI}=\frac{1}{I\times N}\overset{I}{\underset{i=1}{\sum }}\;\overset{N}{\underset{t=1}{\sum }}\;e^{\;j|\varphi_{\mathrm{EEG}}(t,i)-\varphi_{\sin}(t,i)|},$$

where *N* represents the number of samples, $\varphi_{\mathrm{EEG}}(t,i)$ represents the phase value of the filtered EEG time series, and $\varphi_{\sin}(t,i)\;$ represents the phase value of the sinusoidal time series at trial *i* and sample *t*.

### Spectral power calculation

In order to investigate the impact of low-frequency spontaneous activity on the evaluation of brain synchronization to periodicities of duple and triple meter, we calculated for each subject the power spectrum over the stimulation period to examine the spectral power across different frequency bands, including the low-frequency range. We then compared it across participants ranging from younger to older age. We first applied a discrete Fourier transform to the nonoverlapping EEG trials. Then, we averaged the power across all trials to obtain smooth power spectra over all the electrodes. Finally, the average power within the frequency range of 1–1.7 Hz was calculated and subjected to further statistical analyses.

### Statistical analyses

Statistical analyses were performed in MATLAB (MathWorks), using FieldTrip (Oostenveld et al., 2011) and CircStat (Berens, 2009) toolboxes as well as custom MATLAB codes.

To assess the coupling strength of neural oscillation to periodicities related to either beat or meter structures across participants, we generated surrogate data by randomly shuffling the sinusoidal signals 1,000 times. Subsequently, we calculated the coupling strength between each shuffled signal and the narrow-band filtered EEG signals, resulting in 1,000 surrogate SI values at each frequency of interest and each electrode. These values allowed us to establish the SI chance distribution at the population level. We then compared the observed coupling strengths (SI absolute value) with those corresponding to the surrogate datasets using the complementary error function method (Theiler et al., 1992), which enabled us to identify electrodes where the coupling strength was significantly above chance level (*p* < 0.05). Subsequently, to correct for multiple comparisons, we applied the Benjamini–Hochberg false discovery rate correction (Benjamini and Hochberg, 1995) to adjust the *p*-values of all electrodes. Adjusted value of *p* < 0.05 was considered significant. After correcting for multiple comparisons, we detected spatial clusters with significant coupling strength. The initial threshold for cluster definition and the minimum number of neighbors were set to *p* < 0.05 and three, respectively.

When needed, we employed Bayesian statistics to derive a Bayes factor to provide an unbiased decision criterion concerning the null hypothesis. We used the Akaike information criterion to compute the Bayes factors between the “null” and “effect” hypotheses (Kass and Raftery, 1995). According to this symmetric hypothesis comparison measure, a Bayes factor of ≤0.33 signifies evidence in support of the null hypothesis.

To investigate the evolution of stimulus–brain synchronization with gestational age, we *z*-scored the SI absolute values at the population level at each electrode location. The relation between the *z*-scored values and gestational age was investigated using Spearman correlation [normality was tested using the Lilliefors test (Lilliefors, 1969)]. The relation between the *z*-scored low frequency power and gestational age was investigated using Pearson’s correlation.

To investigate the effect of age on stimulus–brain synchronization at different periodicities, we conducted a 3 × 2 analysis of variance (ANOVA) with frequency as a within-subject factor [with beat (3.33 Hz), duple meter (1.67 Hz), and triple meter (1.11 Hz)] and age as a between-subject factor (<33 wGA vs ≥33 wGA). To investigate the effect of age (<33 wGA vs ≥33 wGA) on low frequency power, we conducted a two-sample *t* test over the average frequency power of 1–3.4 Hz. To assess the variability of the preferred phase of coupling across participants, we conducted the Rayleigh test, which allowed us to evaluate the nonuniformity of the circular histogram. This provided a measure of consistency in the phase of coupling among participants. To investigate the correlation between the preferred phase of coupling and gestational age at birth, a circular–linear correlation was calculated (Berens, 2009).

### Data availability

The stimuli and data supporting the study's findings can be obtained upon reasonable request from the lead contact. Due to parents’ nonconsent to share their data beyond our research consortium, the data are not publicly accessible as per the consent form. The MATLAB code and data matrices are available on GitHub (https://github.com/bsaadatmehr/Early-Rhythm-Development/tree/main).

## Results

### Neural synchronization to the metric hierarchy develops gradually during the third trimester of gestation

To investigate the synchronization between neural activity (measured by EEG) and the rhythmic stimuli at beat and meter frequencies, we evaluated the strength of coupling (stimulus–brain synchronization) between the phase of simulated sinusoidal signals, representing the beat, duple meter, and triple meter of the stimulus, and that of the associated narrow-band filtered neural activity. Figure 1 shows the topographical scalp distributions of the grand average SI absolute values for beat-related (Fig. 1*B*) and meter-related (Fig. 1*C*) frequencies across all participants. Stimulus–brain synchronization was then compared with the condition where the stimulus signal was randomly permuted to create surrogate data. The SI absolute values were contrasted to the chance level modeled through surrogate data at each electrode location and for each frequency of interest (i.e., 3.33 Hz for beat, 1.67 Hz for duple meter, and 1.11 Hz for triple meter). We observed a frontocentral cluster for the beat frequency (*p* = 0.001), a frontocentral cluster for the duple meter frequency (*p* = 0.03) with a relatively more focal topographical distribution, and a frontocentral cluster for the triple meter frequency (*p* = 0.04).

To examine the relation between gestational age and neural response to the rhythmic hierarchy, we conducted correlation analyses on specified regions of interest (defined as the aforementioned detected clusters) between gestational age (using the full cohort) and coupling strength for each frequency of interest (Fig. 1*D,E*). These analyses revealed that neural synchronization to the metrical hierarchy develops gradually with gestational age for beat- and meter-related frequencies. More precisely, there were significant positive correlations between gestational age and average *z*-scored SI absolute values at beat (Spearman correlation, *ρ* = 0.37, *p* = 0.01), duple meter (Spearman correlation, *ρ* = 0.31, *p* = 0.03), and triple meter (Spearman correlation *ρ* = 0.30, *p* = 0.03) frequencies.

The correlation between the low frequency power over the shared electrodes across the clusters in Figure 1, *B* and *C*, and gestational age (Pearson’s correlation, *p* = 0.85, Bayes factor = 0.12) was not significant, suggesting that, for the cohort studied, low-frequency spontaneous activity did not significantly decrease as gestational age increased. This observation suggests that the increased neural synchronization to the metrical hierarchy was not related to the possible decrease of low frequency power (hence a decrease in “background noise”) with gestational age at birth.

Finally, as a control analysis, we evaluated neural synchronization at frequencies where no synchronization was expected. We selected frequencies unrelated to the beat and meter, falling halfway between the frequencies of interest and absent in the stimulus spectrum (1.389, 2.500, and 3.055 Hz). We applied the same procedure as described for the beat- and meter-related frequencies and did not find any significant neural synchronization at any of the aforementioned unrelated frequencies. In addition, there was no significant correlation between the strength of coupling at the three aforementioned frequencies and gestational age.

### Synchronization to beat and meter periodicities have different developmental timelines

**Figure 2.**
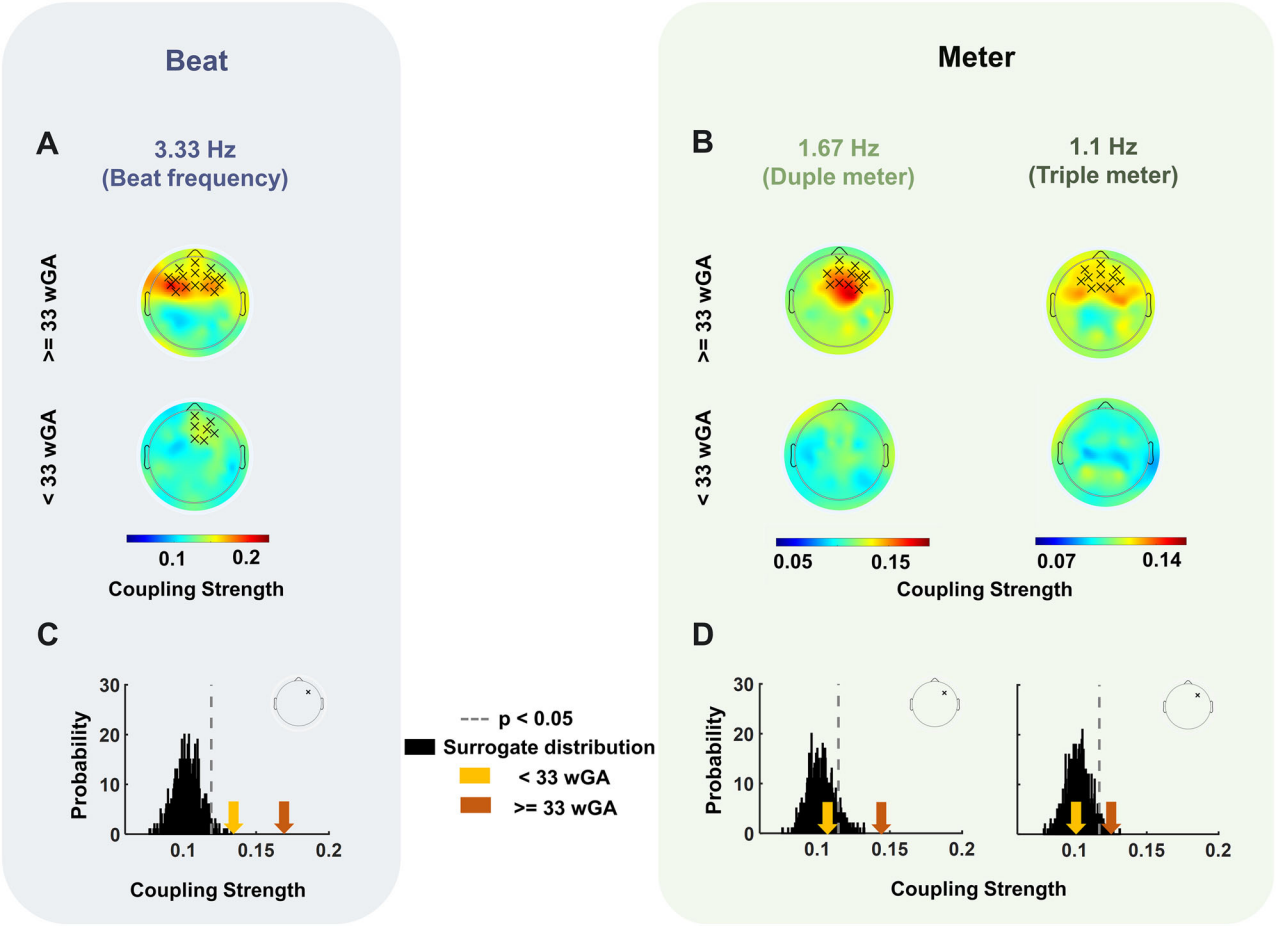
Comparing neural synchronization to beat and meter periodicities. The topographical scalp distributions of the average SI absolute values for both beat- (***A***) and meter-related (***B***) frequencies, separately averaged across the younger and the older age groups. The black crosses overlaid on the topographical distributions represent clusters where the coupling strength was significant: frontocentral clusters for the beat (***A***, *p* < 0.001), the duple meter (***B***, left, *p* = 0.043), and the triple meter (***B***, right, *p* = 0.041) frequencies for the older group (***A***, ***B***, top row) and a significant frontal cluster (smaller in size compared with that of the older group) only for the beat frequency (***A***, *p* = 0.042) for the younger group (bottom row). ***C***, ***D***, Comparing the observed brain–stimulus SI absolute values to chance level for a single electrode (shown over the head map). The black distribution represents 1,000 surrogate SI values, with a significance threshold set at *p* = 0.05. For the beat frequency, coupling strength is on average above chance level for both age groups (***C***), while for meter-related frequencies, the older group showed above chance level responses, whereas the responses of the younger group fell below the chance level (***D***). Extended data on more individual electrodes are presented in Extended Data Figure 2-1.

For further evaluation of the impact of neurodevelopment on neural synchronization to the rhythmic hierarchy, participants were divided into two age groups by median split: <33 wGA (younger preterm, *n* = 23) and ≥33 wGA (older preterm, *n* = 23) based on their age at the time of recording. We performed the same analyses for each age group separately. For the older preterms, significant clusters emerged at beat- (Fig. 2*A*) and meter-related (Fig. 2*B*) frequencies when contrasted to chance level as modeled through surrogate data created for the older group only. This is similar to the results of the full cohort. Precisely, we observed significant frontocentral clusters for the beat (*p* < 0.001), the duple meter (*p* = 0.04), and the triple meter (*p* = 0.04) frequencies. However, for the younger preterms, a significant frontal cluster (smaller in size compared with that of the older group) emerged only for the beat frequency (*p* = 0.04), with no significant cluster for the duple or triple meter frequencies. Further evaluation of the stimulus–brain SI absolute values at individual electrode positions (Fig. 2*C,D*; Extended Data Fig. 2-1) showed that coupling strength was, on average, significantly above chance level for both age groups for the beat frequency (Fig. 2*C*), with larger coupling strength for the older preterms compared with the younger preterms. However, for both duple and triple meter-related frequencies, coupling strength was not above chance level for the younger preterms, whereas it was above chance level for the older preterms (Fig. 2*D*). A one-tailed one-sample *t* test evaluating whether the coupling strength was larger than that corresponding to the 95% confidence interval for the surrogate data at all the electrode locations was not significant (for all the electrodes *p* > 0.1; Bayes factor < 0.33).

10.1523/JNEUROSCI.0398-24.2024.f2-1 Figure 2-1 **Comparing the brain-stimulus SI absolute values to the chance level at individual electrode positions** (shown over the head map). The black distribution represents 1000 surrogate SI values, with a significance threshold set at *p* = 0.05. For the beat frequency (A), coupling strength is on average above chance level for both age groups, while for meter-related frequencies, the older group shows significance compared to chance level, whereas the younger subjects fall below the chance levels (B). Download Figure 2-1, TIF file.

Furthermore, the ANOVA on the strength of coupling averaged across the shared electrodes across the detected clusters presented in Figure 1, *B* and *C*, revealed significant main effects of age (*F*_(1,44)_ = 10.62, *p* = 0.002, $\eta_{p}^{2}=0.19$) and frequency (*F*_(2,88)_ = 6.41, *p* = 0.003, $\eta_{p}^{2}=0.13$). However, the interaction between frequency and age was not significant (*p* = 0.78, $\eta_{p}^{2}=0.005$, Bayes factor = 0.52). Note that the Bayes factor testing for an absence of interaction provided only weak evidence (BF = 0.52), suggesting that this observation has to be treated with caution.

The two-sample *t* test conducted over the average low frequency power over the same electrodes showed that the difference between the spectral power in the range covering the frequencies of interest was not significant between the two age groups (*p* = 0.59, Bayes factor = 0.32), again suggesting that the age-related differences in synchronization to meter periodicities were not due to decreased noise with age.

### Synchronization to the beat periodicity becomes more stable with gestational age

**Figure 3.**
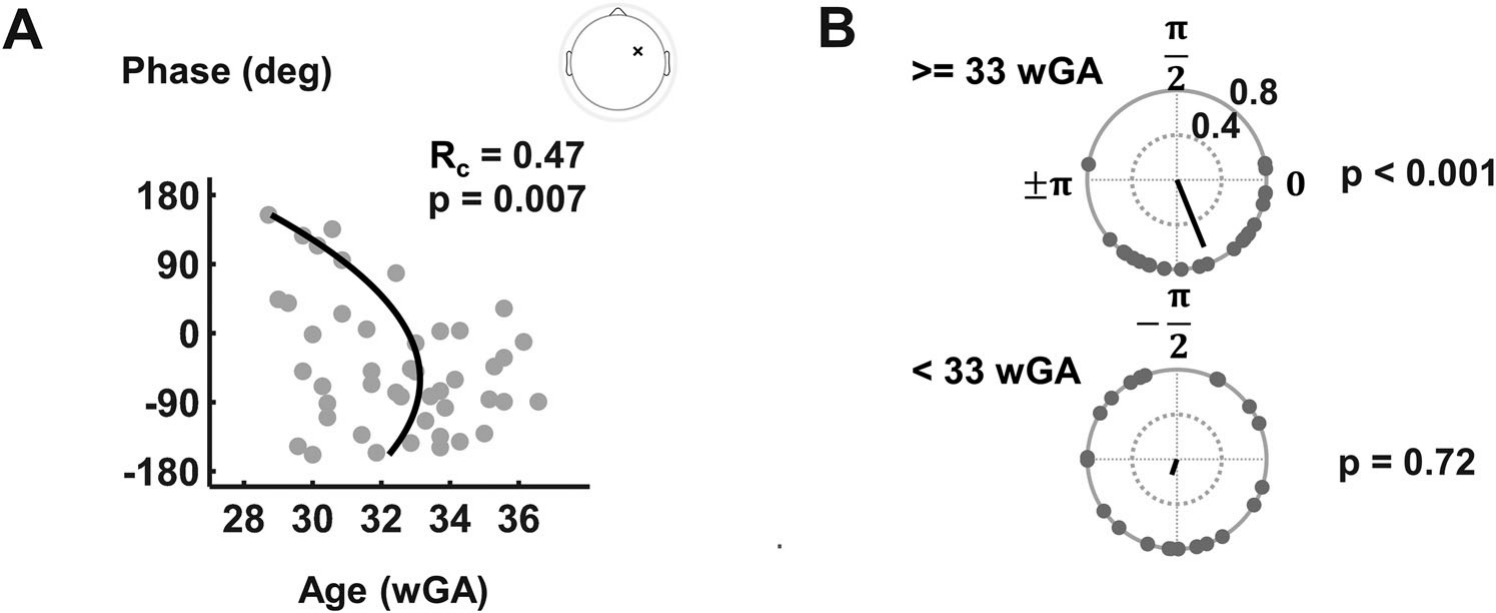
Neural oscillations coupling phase with the beat periodicity. ***A***, Circular–linear scatter plot of the evolution of the phase of coupling at the beat frequency with gestational age on one sample electrode. Circular–linear correlation analysis between the phase of synchronization and gestational age was significant (*R_c_* = 0.47, *p* = 0.007). To further visualize nonlinear circular–linear relationship, a quadratic fit is shown as a black solid curve. ***B***, Comparing the phase distribution between the older (top) and younger (bottom) age groups. The black line illustrates the average phase across subjects within each age group. Rayleigh test verified as significant for the older group (*z* = 9.84, *p* < 0.001), while it was not significant for the younger group (*p* = 0.72). Extended data on more individual electrodes are presented in Extended Data Figure 3-1.

We further evaluated the coupling phase of the neural oscillations to the periodicity corresponding to the beat. This analysis was performed only at the beat frequency and over the frontocentral electrodes as the strength of neural synchronization was significant for the younger premature neonates only at the beat frequency and over these electrodes. We studied the evolution of the coupling phase with gestational age. Circular–linear correlation analyses revealed significant circular–linear correlations between the coupling phase and gestational age (*R_c_* = 0.47, *p* = 0.007 for the sample electrode in Fig. 3; Extended Data Fig. 3-1). Visual inspection of the circular–linear scatter plot (Fig. 3*A*) revealed that with increasing gestational age, the variability of the coupling phase among participants decreased and the phase values became concentrated near zero, suggesting a small-lag phase coupling to the periodicity corresponding to the beat at later gestational weeks (Fig. 3*B*). Rayleigh tests confirmed significance for the older preterms (*z* = 9.84, *p* < 0.001), but not for the younger preterms (*p* = 0.72) at the sample electrode (see Extended Data Fig. 3-1 for more results).

10.1523/JNEUROSCI.0398-24.2024.f3-1 Figure 3-1 **Neural oscillations coupling phase with the beat periodicity at individual electrode positions**. Individual phase coupling analysis at beat frequency focusing on of the electrodes where the coupling strength is significant in both age groups (shown over the head map). (A) Circular–linear correlation analyses between the coupling phase and gestational age. To further visualize nonlinear circular-linear relationship a quadratic fit is shown as a black solid curve. (B) Comparing the distribution of coupling phase between the older (top) and younger (bottom) age groups. The black line illustrates the average phase across subjects within each age group. The more concentrated phase coupling distribution among older subjects is visually depicted and confirmed as the Rayleigh test indicated significance over all three specific electrodes (*p* < 0.05). Download Figure 3-1, TIF file.

## Discussion

In this cross-sectional study, we exposed premature neonates between 28 and 36 wGA to an auditory rhythm with nested regularities, resulting in stimulus energy peaks at the beat frequency, as well as duple meter (groupings of two beats) and triple meter (groupings of three beats) frequencies while we recorded dense array EEG. With increasing age, the phase differences between the beat events and neural responses became less variable among participants and converged on small phase differences between stimulus and neural responses, similar to those observed in adults (Tal et al., 2017; Doelling et al., 2019). Correlational analyses showed that neural synchronization to the beat and meter frequencies became gradually stronger with increasing gestational age. An important impact of gestational age was observed when we evaluated neural synchronization in younger premature neonates recorded before 33 wGA and older premature neonates recorded at or after 33 wGA. For the older preterms, we replicated our previous findings of significant neural synchronization at beat and meter frequencies compared with chance level and the frontocentral topographical distribution of the neural response (Edalati et al., 2023). However, for the younger preterms, only neural synchronization at the beat frequency was observed to be significantly different from chance level. In other words, both age groups showed significant neural synchronization to the fast periodicity related to the beat, whereas only the older group showed significant neural synchronization to the slower meter frequencies related to the neural representation of beat groupings. We showed that there were no significant decreasing trends in the noise floor EEG power spectrum over the low-frequency range, so it is unlikely that these age-related results were a consequence of greater noise at lower frequencies in the younger preterm.

Direct comparison of the strength of neural synchronization to beat and meter periodicities after grouping participants into younger and older premature neonates showed an increased neural synchronization in the older group compared with the younger group. We did not find a significant interaction between age and frequency (beat, duple, triple). While this lack of significant interaction might suggest that neural synchronization to beat and meter develop at similar rates, any conclusions based on this lack of interaction need to be tempered by the finding that neural synchronization in the younger infants was at chance levels, making a lack of interaction difficult to interpret. Therefore, we cannot draw conclusions as to whether neural tracking of beat and meter have similar or different developmental rates. Future studies on larger cohorts should address this limitation and investigate this question in more detail.

Two factors could drive the modulation of the response by gestational age at birth: (1) the maturation of neural circuit interactions and (2) the duration of exposure to the intrauterine sound environment, which includes prominent rhythmicities in the maternal heartbeat and voice, and rhythmic stimulation through other modalities, such as vestibular and tactile, that the infant experiences as the mother locomotes rhythmically, for example.

### The impact of neural maturation on neural coding of rhythm

Neural models have been proposed to simulate how the human brain might learn to process complex auditory rhythms containing multiple frequencies. In particular, nonlinear oscillation-based models show considerable promise (Large et al., 2023). In these models, high-frequency oscillators have faster dynamics than low-frequency oscillators so that the resonance width (similar to the bandwidth of a filter) is constant when measured on a logarithmic frequency scale (the so-called constant-Q behavior). With faster dynamics at high frequencies, the network “learns” the relationships between high frequencies earlier than low frequencies (Kim and Large, 2021; Tichko et al., 2022). This might in part explain the developmental enhancement of neural synchronization to slower frequencies. In addition, in these models, the learning of the phase relationships between the frequencies of the exogenous rhythm and the endogenous oscillations depends on the plasticity of the synaptic connections in networks composed of excitatory and inhibitory populations (Large and Snyder, 2009; Pittman-Polletta et al., 2021). Given the modulation of excitatory–inhibitory balance during early neurodevelopment (Hanganu-Opatz et al., 2021; Chini et al., 2022), we might hypothesize that the neocortical development of excitatory–inhibitory interactions may lead to the strengthening of neural responses to slower meter-related frequencies during late gestation. However, these hypotheses are speculative for now as it is based on separate observations and not directly linked variables and remain for future work to investigate.

### Neural coding of rhythm and early auditory experience

Previous research has suggested that full-term newborns are quite proficient at the neural encoding of metrical structure (Winkler et al., 2009), prosodic boundaries (Abboub et al., 2016), and statistical regularities in auditory streams (Fló et al., 2019, 2022a; Moser et al., 2020; Panzani et al., 2023)—capacities that require temporal integration of events in auditory sequences—but there is little research on when these capabilities emerge prenatally. Our correlation results show that sensitivity to metrical structure in rhythm patterns emerges gradually during the third trimester, and we hypothesize that a general capacity for extracting regularity in patterns may emerge gradually during the months before full-term birth. Language structure also involves hierarchical temporal levels, in which phonemes group to form syllables, and syllables group to form words, with the particular rules governing this grouping structure differing somewhat across languages. Consistent with the hypothesis that learning such temporal structures emerges progressively over the third trimester, Moser et al. (2021) tested fetuses between 25 and 40 wGA and showed with magnetoencephalography that hierarchical rule learning in syllabic sequences was only present after 35 wGA. This, together with our results, leads to the hypothesis that capacities for processing hierarchical temporal structure in music and language may go through a similar gradual prenatal emergence.

This now raises the question about the potential role of prenatal experience in the emergence of neural networks for processing the temporal structures of auditory sequences. There is some evidence that the auditory processing of full-term newborns is affected by their prenatal experience. For example, full-term newborns can discriminate their native language from a rhythmically different language (Moon et al., 1993; Nazzi et al., 1998; Byers-Heinlein et al., 2010) and can discriminate patterns with well-formed versus ill-formed prosody if exposed to such patterns prenatally (Abboub et al., 2016). In addition, their crying patterns depend on the maternal native language (Mampe et al., 2009). However, the prenatal auditory environment during the last trimester of gestation differs greatly from the auditory environment of prematurely born infants in the NICU. While characteristics of the NICU auditory environment have been documented (Wachman and Lahav, 2010), the differing effects of the womb and NICU environments on brain development are understudied. In the present sample, we did not have the opportunity to record the younger preterms of our sample a second time at the equivalent corrected age as that of the older preterm group. Further research needs to evaluate the impact of extrauterine neurodevelopment on the characteristics of the neural response to rhythmic stimulation. We would hypothesize that differences may exist between the two groups if the early preterm neonates were tested again at the equivalent corrected age. Evidence from animal models of early neurodevelopment has shown that modifications of bottom-up sensory input influence neural circuit development (Ibrahim et al., 2021). In humans, prenatal musical and linguistic auditory experience alters perception shortly after birth (Moon et al., 2013; Arenillas-Alcón et al., 2023). Collectively, the evidence suggests an impact of altered auditory experience on development in prematurely born infants, but further research is needed to fully understand the impact on the complex pattern perception needed for speech and music.

### Early neural coding during sleep

A recent study has reported that the neural response to meter, which is present during wakefulness, is attenuated in sleeping adults (Sifuentes-Ortega et al., 2022). The premature infants in our study were tested while sleeping [as in Edalati et al. (2023) and Fló et al. (2019)]. It is important to point out that a major distinction exists between adults and neonates (late preterm and full term) in the capacity to perform complex neural coding and computing during sleep (Dehaene-Lambertz and Spelke, 2015), with sleeping newborns showing neural capacities for coding aspects of the auditory world related to music and speech that are less evident in adults during sleep (François et al., 2017; Háden et al., 2020, 2024; Kujala et al., 2023). These differences in neural capacities during sleep might be due to differences in the organization of sleep–wake structure and mechanisms. Prominent electroencephalographic features of nonrapid eye movement sleep emerge around 2 months after full-term birth (Jenni et al., 2004). In addition, sleep spindles, which play a refractory role in thalamic inputs (Antony et al., 2018), first emerge around the second month after full-term birth (Friedrich et al., 2019). Starting at approximately 28 wGA, sleep comprises only two stages, quiet sleep (∼40% of a sleep cycle at birth) and active sleep (50–60% of a sleep cycle at birth) with many microarousal periods within and between sleep stages (Scher, 2008). The short periods of wakefulness are followed by active sleep, which can be considered equivalent to rapid eye movement sleep in adults. In adults, learning in general (Andrillon et al., 2016) and neural response to meter in particular (Sifuentes-Ortega et al., 2022) have been shown to occur during rapid eye movement sleep. This leads to the hypothesis that during the very early stages of neurodevelopment, the fetus or the neonate may learn and consolidate more efficiently during sleep than later in development. In future studies, it would be interesting to address the impact of vigilance states and their evolution with neurodevelopment on the neural coding of temporal sequences in premature neonates.

### Limitations of the study

In the current study, we cannot separate the evoked potential responses to isolated tones and oscillations entrained by beat periodicity. Thus, the response at the beat tempo may include both evoked potentials and synchronization to the periodicity. Previous cross-sectional studies have shown that auditory evoked potential changes with age before term between 29 and 34 wGA (Daneshvarfard et al., 2019). Our finding that the phase coupling between the stimulus and brain response develops from highly variable phase differences in the younger neonates, to relatively more stable phase differences in older neonates, suggests that the neural response to the beat evolves toward coding of the periodicity in the stimulus. Furthermore, the lack of significant response to meter groupings in the younger neonates suggests that the developmental differences are not driven solely by differences in evoked responses. However, it remains possible that younger preterms might better encode periodicities at faster tempos, so it remains for future research to test whether preterms at this age might show neural responses to meter groupings for faster presentation rates.

Our protocol included only one rhythmic pattern, which limits interpretations regarding the development of neural responses specific to the metrical hierarchy of our stimulus. Although the results are consistent among participants, the generalizability of the results should be further tested in future studies using stimuli characterized by different metrical hierarchies as well as by comparing responses to stimuli with both strong and weak periodicities. Converging evidence across such studies will allow a more solid interpretation of the development of the neural coding of rhythm and its underlying beat and meter structures.

## Conclusion

The auditory world of the fetus is rich in rhythmic information with omnipresent rhythmic maternal sounds, rhythmic stimulation through other modalities, and extrauterine music and speech reaching the fetus through the maternal tissues. Our study of newly born premature infants at various GAs provides the first evidence for the development of the neural coding of rhythm during the very early stages of auditory neurodevelopment, when the neural response can first be recorded in humans from the first waves of neurons arriving at the cortical targets and the thalamic afferents entering the cortical plate starting from approximately 28 wGA. Our novel finding of the fast early development of neural capacities for periodicity tracking between 28 and 36 wGA highlights that rhythmic processing may be a guiding principle during early cortical maturation. The NICU environment in which premature infants develop is noisy and lacks the rhythmic stimuli omnipresent in the uterus, such as the mother's heartbeat and voice. Future work should now also investigate the neurodevelopmental consequences of these perturbations in the early exogenous information reaching prematurely born infants as they develop in the NICU, on rhythmic, language, and musical development.
