# Supplementary figures and images for: Auditory Rhythm Encoding during the Last Trimester of Human Gestation: From Tracking the Basic Beat to Tracking Hierarchical Nested Temporal Structures
Source: J Neurosci. 2024 Dec 23;45(4):e0398242024. doi: 10.1523/JNEUROSCI.0398-24.2024 (PMC11756625; doi:10.1523/JNEUROSCI.0398-24.2024)

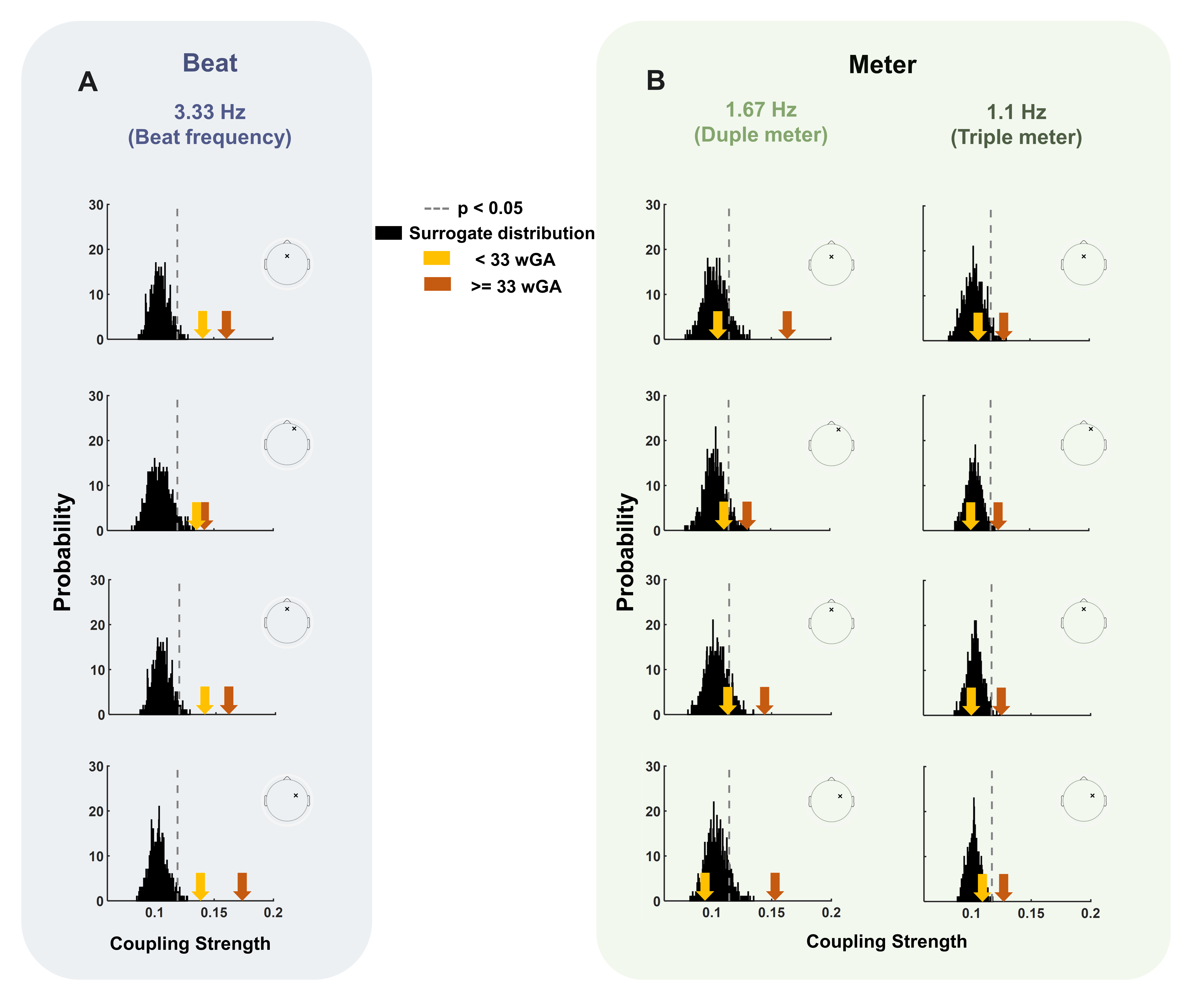

Supplement: Figure 2-1 — Comparing the brain-stimulus SI absolute values to the chance level at individual electrode positions (shown over the head map). The black distribution represents 1000 surrogate SI values, with a significance threshold set at p = 0.05. For the beat frequency (A), coupling strength is on average above chance level for both age groups, while for meter-related frequencies, the older group shows significance compared to chance level, whereas the younger subjects fall below the chance levels (B). Download Figure 2-1, TIF file. [file jneuro-45-e0398242024-s001.tif]

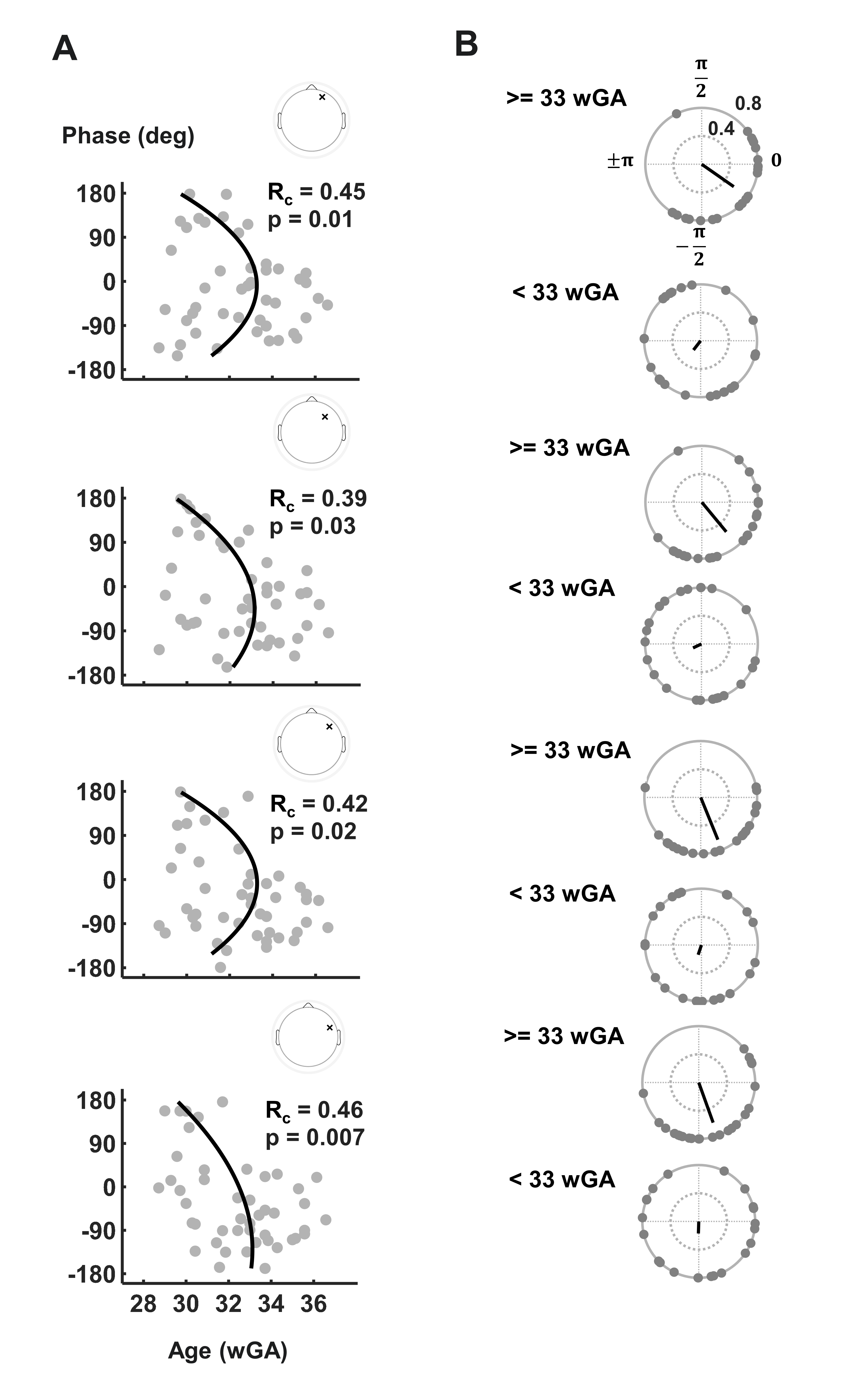

Supplement: Figure 3-1 — Neural oscillations coupling phase with the beat periodicity at individual electrode positions. Individual phase coupling analysis at beat frequency focusing on of the electrodes where the coupling strength is significant in both age groups (shown over the head map). (A) Circular–linear correlation analyses between the coupling phase and gestational age. To further visualize nonlinear circular-linear relationship a quadratic fit is shown as a black solid curve. (B) Comparing the distribution of coupling phase between the older (top) and younger (bottom) age groups. The black line illustrates the average phase across subjects within each age group. The more concentrated phase coupling distribution among older subjects is visually depicted and confirmed as the Rayleigh test indicated significance over all three specific electrodes (p < 0.05). Download Figure 3-1, TIF file. [file jneuro-45-e0398242024-s002.tif]
